# Whole-cell morphological properties of neurons constrain the nonrandom features of network connectivity

**DOI:** 10.1186/1471-2202-16-S1-O7

**Published:** 2015-12-18

**Authors:** Jugoslava Aćimović, Tuomo Mäki-Marttunen, Marja-Leena Linne

**Affiliations:** 1 Department of Signal Processing, Tampere University of Technology, Tampere, Finland; 2 Institute of Clinical Medicine, University of Oslo, Oslo, Norway

## 

We addressed the principles of micro-level organization of neuronal circuits and explored how the neuronal morphology constrains this organization. Several studies have demonstrated the non-trivial properties of the network connectivity using in vitro recordings from multiple neurons [1-3], yet it is unclear to what extent this structure reflects reorganization caused by synaptic plasticity, and what is imposed by the morphological constraints. Two recent articles explored this issue using the simulated neural circuits and demonstrated the specific structural properties in those circuits [4,5].

We analyzed a model that emphasizes the role of single-cell morphology, a homogeneous population of neurons in a planar space without boundaries. Each neuron is composed of two displaced neurite fields defined on the limited support. A neurite field describes the likelihood of finding a neurite segment at a certain point in the plane. Using a proximity criterion (Peters' rule) the expected number of potential synapses is estimated between each pair of neurons. Alternatively, this number can be estimated from the realistic morphology of a simulated neuron, or from the morphologies reconstructed from in vitro/in vivo recordings. The number of potential synapses depends on the axon-dendrite distance, which leads to a definition of the expected radius. An axon-dendrite pair that is expected to form at least one synapse must be on a distance not larger than the effective radius. All considered statistical measures of network connectivity are expressed as the functions of the effective radius normalized with the neuron size. In this study, we considered the standard graph theoretic measures of network connectivity, the motif counts, clustering coefficient, path length, and small-world coefficient. It has been demonstrated that they have a significant impact on the population activity in simulated networks [6].

Changing the normalized effective radius from small (<0.3) to big (>10) we vary the network properties between the two extremes. For the small values of the effective radius, the networks favor unidirectional connections and sparse local connectivity. The clustering coefficient and the path length are similar to those obtained in uniform random networks, i.e. in the networks independent of topology. For the large values of the effective radius, the local connectivity is dense with the majority of bidirectional connections. As the normalized effective radius increases, the clustering coefficient increases towards the values obtained for the networks with dominant local connectivity, while the path length remains close to the one of the uniform random networks. The normalized effective radius on the interval 1-2, provides the biggest variability of connectivity patterns and the optimized properties relevant for the information transfer.

## Conclusions

We present a theoretical framework that relates neuromorphology with the connectivity in neuronal circuits, and that can be solved analytically. The normalized effective radius was found to be the key morphological property that dominantly affects considered connectivity measures. By tuning it we can obtain the networks with the biggest variability of local connectivity patterns. At the same time, those networks acquire the key characteristics of the small-world networks, known to optimize the information transfer.
